# Survival Analysis of Cervical Cancer Patients: A Case Study of Bhutan

**DOI:** 10.31557/APJCP.2021.22.9.2987

**Published:** 2021-09

**Authors:** Ugyen Tshewang, Pairote Satiracoo, Yongwimon Lenbury

**Affiliations:** 1 *Department of Mathematics, Faculty of Science, Mahidol University, Rama 6 Rd, Bangkok, Thailand. *; 2 *Centre of Excellence in Mathematics, Perdo, Thailand. *

**Keywords:** Cervical cancer, Kaplan-Meier model, Cox proportional hazards model, Survival analysis

## Abstract

**Objective:**

The purpose of the study is to identify the risk factors such as age, the stage of patients based on the International Federation of Gynecology and Obstetrics system (FIGO stage) and treatment type, and their effect on the survival of cervical cancer patients receiving treatments at Jigme Dorji Wangchuck National Referral Hospital (JDWNRH) in Bhutan between January 2014 and December 2019.

**Methods:**

In this retrospective study, all 357 women diagnosed with cervical cancer were included. Kaplan-Meier model was applied to estimate survival, and the log-rank test was performed to compare survival distributions between subgroups stratified by each of the risk factors. Baseline demographics, cervical cancer stages, and treatment options were analyzed as factors and predictors of survival by Cox proportional hazards model.

**Results:**

The overall estimated 1- to 5-year survival rates are 82.1% (95% CI: 77.8-86.7), 75.6% (70.4-81.1), 65.2% (58.2-73.0), 62.3% (54.7-70.9) and 55.4% (44.9-68.3). The results reveal that age group, FIGO stage, treatment, and frequency of hospital visits are significant factors affecting the survival of cervical cancer patients in Bhutan. Patients aged >45 years increases the risk of dying (HR: 2.1, 95% CI: 1.2-3.9) compared to the young age group (≤45 years). Treatment types other than surgery only are significantly associated with an increased risk of mortality in patients with cervical cancers. The more frequency of hospital visits also reduces the risk of dying (HR: 0.1, 95% CI: 0-0.3). FIGO stage IV is the most significant risk factor for mortality with a hazard ratio of 6 (95% CI: 2.1-17.6).

**Conclusion:**

The five-year survival rate of cervical cancer patients in this study was low. Late diagnosis of cervical cancer appears to be mainly associated with a higher risk of dying. The results provide valuable information for further research and policymaking in the prevention and management of cervical cancer.

## Introduction

Cervical cancer is widely considered one of the deadliest diseases among women globally, as well as in small countries such as Bhutan. The financial costs of cancer are high for both the person with cancer and for society as a whole. One of the major costs of cancer is cancer treatment, but a lack of health insurance is recognized as an important barrier that prevents many from getting optimal health care (World Cancer Research Fund International). According to the American Institute for Cancer Research, cervical cancer is the fourth most commonly occurring cancer in women and the eighth most commonly occurring cancer overall. Risk assessment is one of the powerful tools that provide a rational framework for designing and managing health insurance provision (American Cancer Society).

In Bhutan, cervical cancer is the first most common cancer among women with 82 deaths recorded between 2014 to 2018. About 70% of the total female population in the reproductive age group is at risk of developing cervical cancer in Bhutan (Bhutan Broadcasting Service Corporation Ltd., 2019). It is estimated that there were 570,000 new cases of cervical cancer worldwide and about 311,000 women died from the disease in 2018 (Arbyn et al., 2020). Bhutan was ranked 101st globally in terms of cervical cancer status. An age-standardized incidence rate of cervical cancer in Bhutan was approximately 13 cases per 100,000 person-years, which is one of the highest among Asian countries (Tshomo et al., 2014). 

The vulnerable age of developing cervical cancer in females is at the age of 15 years and older. Cervical cancer stands as the first most frequent cancer death among women in Bhutan between 15 and 44 years of age (ICO/IARC HPV Information Centre, 2019). The Royal Government of Bhutan spends nearly NU 200,000 for each patient undergoing cervical cancer treatment leading to a huge burden on the country (Bhutan Broadcasting Service Corporation Ltd., 2019).

Early diagnosis of cancer generally increases the chances for successful treatment. Cervical cancer is one of the most successfully treatable forms of cancer, as long as it is detected early and managed effectively. The late or advanced-stage cancer can also be controlled with appropriate treatments and palliative care. The biggest challenges that Bhutan faces at present is that the women never come forward and volunteer for the national screening programme. However, the Royal Government of Bhutan has initiated a national human papillomavirus (HPV) vaccination program for which girls aged 12 are offered the HPV vaccines to prevent HPV infection and HPV-associated cervical cancer in future. Bhutan is one of the first countries in Asia to start a nationwide vaccination programme to fight against human papillomavirus (HPV), despite being one of the economically developing nations. In 2010, Bhutan had estimated to achieve 92% vaccination coverage among girls aged between twelve to eighteen years old (Baussano et al., 2014; Tshomo et al., 2014). However, it has remained a continuous major threat to women’s lives

The purpose of the study is to identify the risk factors such as age, stage of the patient, and treatment type, and their effect on the survival of cervical cancer patients receiving treatments at Jigme Dorji Wangchuck National Referral Hospital (JDWNRH) in Bhutan. 

To estimate the survival function of patients, we utilize the Kaplan-Meier (KM) method. The Kaplan–Meier estimator, according to (Kaplan and Meier, 1958), is also known as the product limit estimator, which is a non-parametric statistic mainly utilized to find estimates for the survival function based on lifetime data. In medical research, researchers frequently use this method to measure the fraction of patients surviving for a certain length of time after receiving treatment. Moreover, the Kaplan-Meier procedure is a useful method to estimate time-to-event models when there are censored cases present. The Kaplan-Meier model involves the estimation of conditional probabilities at each point in time point when an event takes place and the calculation of the product-limit of those probabilities to arrive at the estimate of the survival rate at each time point. An example of applications of this method to cancer research can be seen in (Geng et al., 2017), where a mathematical model was developed to predict Kaplan-Meier survival curves for chemotherapy combined with radiation in patients suffering from lung cancer with non-small cells to design clinical trials.

To examine the effects of several prognostic variables on the patients’ survival, we will perform the univariate and multivariate Cox regression analyses. Cox regression, sometimes called the proportional hazards regression, is a method that researchers frequently used to investigate the impacts which several variables may have upon the time it takes for a specified event to occur. In terms of an outcome such as death, this procedure is known as Cox regression for survival analysis. Though the method does not assume any particular “survival model”, it is not truly nonparametric because no assumptions are made that the effects of the predictor variables upon survival are constant over time and are additive in one scale. Example of applications of Cox regression and survival analyses can be seen in (Mascarello et al., 2013; Saikia and Barman, 2016; Dankner et al., 2019; Khalkhali et al., 2019). In (Dankner et al., 2019), a discrete form of weighted cumulative metformin exposure was utilized to discover the association between metformin and cancer incidence. The authors implemented a time-dependent covariate Cox model which was adjusted for treatment with other glucose-lowering medications, together with the factors of age, sex, ethnic background, socioeconomic status. For bladder and lung cancer patients, the smoking habit was also considered, while the effect from parity was included for breast cancer patients. 

## Materials and Methods

*Study Participants*

Jigme Dorji Wangchuck National Referral Hospital (JDWNRH), situated in the capital city, namely Thimphu, keeps the records and updates the patients’ information suffering from any diseases. The patients included in this study are those whose deaths were due to cervical cancer from January 2014 to December 2019. Therefore, the study includes 357 cervical cancer patients. The patients are classified into two age groups: 138 patients in the young age group (≤45 years) and 219 in the older age group (>45 years).

*Risk factors*

The variables including age, FIGO stage (the stage based on the International Federation of Gynecology and Obstetrics system), treatment center, treatment type, occupation, education, ethnic group, and frequency of hospital visits, were included to figure out the risk factors affecting the survival time. Some other risk factors like the number of partners, age of first pregnancy, current smoking habit, history of pap smear and histopathologic tumor characteristics were absent in the data. To discover their significance to the survival time, the accessible variables have been used.

The characteristics of patients treated at JDWNRH are given in Table 1, where the FIGO stages I to IV can be defined briefly as follows, referring the readers to the American Cancer Society website for more details. Stage I corresponds to the stage in which cancer is confined to the organ of origin, stage II corresponds to the stage in which invasion of surrounding organs or tissue is observed, stage III corresponds to the stage in which cancer has spread to distant nodes or tissue within the pelvis, and stage IV corresponds to the stage in which we observe distant metastasis(es).

*Statistical Analysis*

The Kaplan-Meier (KM) method is used to estimate the survival function of patients and to calculate 1- to 5- year survival statistics. Kaplan-Meier estimated survival curves have been constructed for various categories and compared using the log-rank test. To examine the effects of several prognostic variables on the patients’ survival, the univariate and multivariate Cox regression analyses are performed. Stepwise Cox proportional hazards model has also been utilized to identify potential significant factors influencing survival and the best fit model based on the Akaike Information Criterion. Statistical analysis is performed using R software from the R Project for Statistical Computing (version 3.6.0). A p-value < 0.05 is considered to be statistically significant.

## Results

The baseline characteristics of 357 patients grouped into different categories are shown in Table 1. The mean and standard deviation of the patients’ age are 50.5 years and 13.3 years, respectively. The patients are classified into two age groups: 138 patients in the young age group (≤45 years) and 219 in the older age group (>45 years).

According to the FIGO stages, 27.5% are in stage I, 20.4% in stage II, 12.6% in stage III and 3.1% in stage IV. There are 130 patients (36.4%) with unknown cervical cancer stage. Regarding the frequency of hospital visits classified into once and twice, there are 68.1% and 31.9%, respectively.

The follow-up duration of the study is 6 years, from January 2014 to December 2019. The mean follow-up time is 1.5 years. During the study period, out of 357 cervical cancer patients, 81 patients died which is 22.7% of the total number of patients under study. Regarding treatment categories, patients are grouped into four subgroups: surgery only, radiation and/or chemotherapy, surgery combined radiation and/or chemotherapy, and palliative treatment. The detailed information is presented in Table 1.

**Figure 1:**
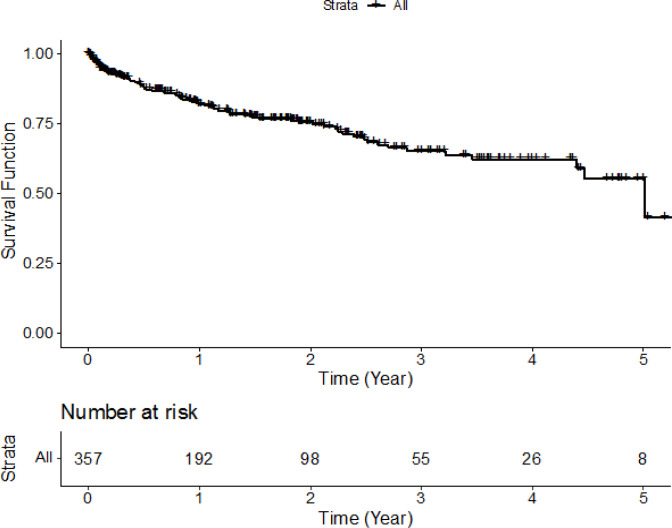
Kaplan-Meier Survival Curve of 357 Cervical Cancer Patients

The Kaplan-Meier estimated survival curve for cervical cancer patients is displayed in Figure 1. The overall estimated 1- to 5-year survival rates are 82.1% (95% CI: 77.8-86.7), 75.6% (70.4-81.1), 65.2% (58.2-73.0), 62.3% (54.7-70.9) and 55.4% (44.9-68.3), respectively. It indicates that the life span of more than 50 women out of every 100 patients diagnosed with cervical cancer would be 5 years or more if the cause of death is not from other diseases. 

**Figure 2:**
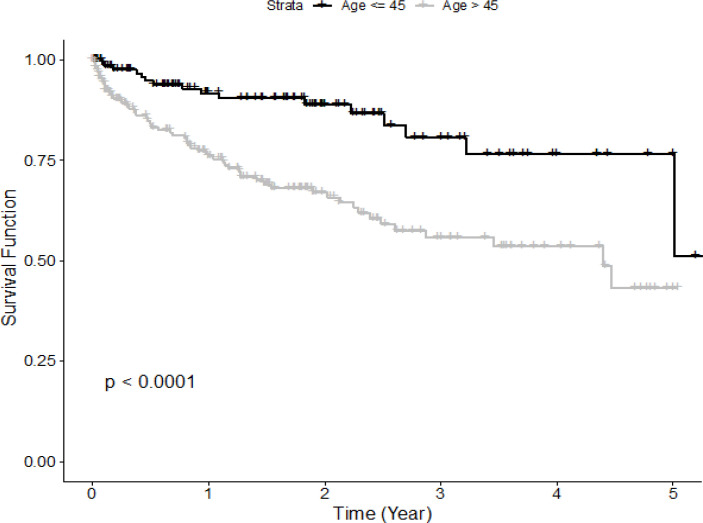
Kaplan-Meier Survival Curves of Cervical Cancer Patients by Age

**Figure 3:**
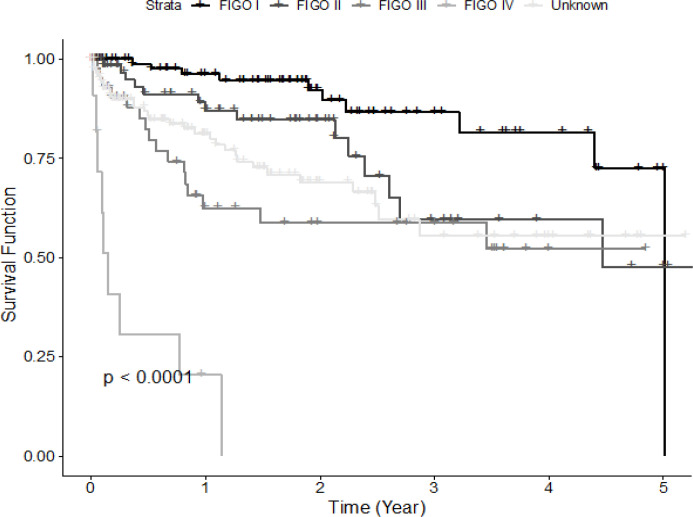
Kaplan-Meier Survival Curves of Cervical Cancer Patients by FIGO Cancer Stage

Figure 2 provides a comparison of the survival functions between different age groups. It shows that patients aged ≤45 years have a better survival rate than patients aged > 45 years. In Figure 3, Patients with early stages (stages I and II) have a longer life expectancy than the diseased people with advanced stages (stages III and IV). If cervical cancer is diagnosed at the early stages it can be treated and has a higher survival rate. Stage I and II patients have survival rates of 96% and 87% at 1 year, 87% and 60% at 3 years, and 73% and 48% at 5 years, respectively. The 1- and 3- year survival rates of the subgroup with stage III are 63% and 59%, respectively. Patients with stage IV has a significantly worst 1-year survival rate from cervical cancer death of 20%.

In Table 2, the log-rank test (p-value < 0.001) also identifies two additional prognostic variables, treatment type and frequency of hospital visits, that contribute to the survival difference. Patients treated with surgery only and patients who have visited the hospital twice have significantly better survival compared to those from the other subgroups in the same categories. The factors including treatment center, occupation, education and ethnic group do not contribute to the survival difference.

Multivariate Cox regression analysis to determine predictive factors with regard to survival is shown in Table 3. Risk factors that have been considered in the Cox proportional hazards model for the survival rate of cervical cancer patients are age group, FIGO stage, treatment center, treatment type, occupation, education, ethnic group and frequency of hospital visits. All of these prognostic variables included in the Cox model satisfy the proportional hazards assumption. Furthermore, the stepwise Cox proportional hazards model has been utilized to identify the best fit model based on the Akaike Information Criterion. The variables excluded from the final model are occupation, education and ethnic group. The results show that those patients aged >45 years increases the risk of dying (HR: 2.1, 95% CI: 1.2-3.9) compared to the young age group (≤45 years). Treatment types other than surgery only are significantly associated with an increased risk of mortality in patients with cervical cancers. The more frequency of hospital visits also reduces the risk of dying (HR: 0.1, 95% CI: 0-0.3). FIGO stage IV is the most significant risk factor for mortality with a hazard ratio of 6 (2.1-17.6). At univariate analysis FIGO stages II and III are associated with a worse prognosis even if multivariate results do not reach statistical significance at 5% level (HR = 1.2, 95% CI: 0.5-2.8 for stage II, HR = 2.3, 95% CI: 0.9-5.6 for stage III). 

**Table 1:** Baseline Characteristics of 357 Cervical Cancer Patients: Number (%)

Factor	Number	Percent
Age at dianosis		
≤45	138	38.7
>45	219	61.3
FIGO Stage		
FIGO I	98	27.5
FIGO II	73	20.4
FIGO III	45	12.6
FIGO IV	11	3.1
Unknown	130	36.4
Treatment type		
Surgery only	122	34.2
Radiation and/or chemotherapy	179	50.1
Surgery combined radiation and/or chemotherapy	42	11.8
Palliative treatment	14	3.9
Treatment center		
Bhutan	195	54.6
Outside Bhutan	162	45.4
Occupation		
No work	239	66.9
Government service	13	3.6
Corporate employment	7	2.0
Farmer	98	27.5
Education		
No education	273	76.5
Non-formal education	43	12
Primary education	16	4.5
Secondary education	23	6.4
University education	2	0.6
Ethnic group		
Sharshop	142	39.8
Ngalong	114	31.9
Lhotsam	45	12.6
Others	56	15.7
Frequency of hospital visits		
Once	243	68.1
Twice	114	31.9

**Table 2:** Kaplan-Meier Estimated Survival Rates at 1,3 and 5- years among Cervical Cancer Patients (p-value < 0.001)

Factor	Survival rate		
	(95% CI)		
	1 year	3 year	5 year
Age at dianosis			
≤45	92 (87-97)	81 (71-92)	77 (65-91)
>45	76 (70-83)	56 (47-66)	43 (30-62)
FIGO Stage			
FIGO I	96 (92-100)	87 (77-97)	73 (55-96)
FIGO II	87 (79-97)	60 (43-83)	48 (28-83)
FIGO III	63 (49-80)	59 (45-78)	NA
FIGO IV	20 (6-70)	NA	NA
Unknown	81 (74-89)	56 (43-72)	56 (43-72)
Treatment type			
Surgery only	92 (87-98)	85 (77-95)	81 (69-94)
Radiation and/or chemotherapy	80 (74-87)	59 (49-72)	49 (35-69)
Surgery combined RT and/or CT	87 (76-100)	59 (42-83)	44 (23-86)
Palliative treatment	7 (1-47)	NA	NA
Frequency of hospital visits			
Once	76 (71-82)	54 (46-64)	45 (34-58)
Twice	96 (92-100)	96 (92-100)	96 (92-100)

**Table 3:** Relative Risk of Death for Cervical Cancer Patients

Factor	HR	(95% CI)	p-value
Age at dianosis			
≤45	Reference level		
>45	2.1	1.2-3.9	0.014
FIGO Stage			
FIGO I	Reference level		
FIGO II	1.2	0.5-2.8	0.737
FIGO III	2.3	0.9-5.6	0.067
FIGO IV	6	2.1-17.6	0.001
Unknown	1.6	0.8-3.5	0.213
Treatment type			
Surgery only	Reference level		
Radiation and/or chemotherapy	3.1	1.3-7	0.008
Surgery combined RT and/or CT	3.7	1.6-8.6	0.002
Palliative treatment	30.1	12-75.6	<0.001
Treatment center			
Bhutan	Reference level		
Outside Bhutan	0.6	0.3-1.1	0.126
Frequency of hospital visits			
Once	Reference level		
Twice	0.1	0-0.3	<0.001

## Discussion

Cervical cancer is considered one of the life-threatening diseases and major cause of death in the female population globally. In 2018, an estimate of more than half of a million new cases is diagnosed each year, with more than half of them dying yearly worldwide, causing a great deal of burden on the economy. About 85% of the worldwide deaths occur in underdeveloped or developing countries. The majority of which occur in Central and South America, the Caribbean, Sub-Saharan Africa and Southern Asia (Small et al., 2017; Arbyn et al., 2020). Several factors have been reported as important factors influence survival in cervical cancer patients such as low age at marriage, social and cultural factors, the stage of cervical cancer at diagnosis, and tumor size and volume. From the present study of 357 patients, the mean age at the time of diagnosis is 50.5 years with a standard deviation of 13.3 years, and the median age is 49 years. The overall estimated 1, 3 and 5-year survival rates are 82.1%, 65.2% and 55.4%, respectively. According to the recent results, the overall survival rates at 1, 3 and 5 years of 5,859 cervical cancer patients in Malaysia during 2000 and 2015 (Muhamad et al., 2015) were 97%, 85% and 73%, of 109 patients in Azerbaijan during 2004 and 2015 (Khalkhali et al., 2019) were 94%, 79% and 71%, and of 558 patients diagnosed in an extensive screening program in rural India during 2000 and 2013 (Jayant et al., 2016) were 82%, 68% and 60%, respectively. In this present study, the patients diagnosed with cervical cancer in Bhutan have a lower life expectancy than those in the aforementioned reports. The substantially low overall survival rates in this study compared to those countries might be due to the effectiveness of the cancer care system both early detection of the diseases and the effectiveness of treatment.

The results of this study reveal that patients diagnosed at old age are significantly associated with a reduced overall survival rate. This implies that 5-year survival for cervical cancer patients is higher in the young age group and decreases with increasing age. The association between age at diagnosis and survival in cervical cancers has been confirmed by the recent study (Quinn et al., 2019). That study was a large investigational study in the United States of women diagnosed with cervical cancer between the period of 1973 and 2015 using the Surveillance, Epidemiology and End Results (SEER) database. 

The stage at diagnosis shows a crucial association with survival in cervical cancer. Patients with FIGO stage III and IV are 2.3 and 6 times at high risk of mortality compared to those with FIGO stage I. As the stage increases, the possibility of distant tumor metastasis will also increase, which results in a dramatic decrease in the life expectancy of patients. Patients with early stages (stage I and stage II) have a longer life expectancy than the diseased people with advanced stages (stage III and IV). If cervical cancer is diagnosed at the early stages it can be treated and has a higher survival rate. This result is consistent with other findings (Razak et al., 2013; Gurmu, 2018; Khalkhali et al., 2019). It should also be emphasized that there is 36.4% of patients with unknown FIGO stages in this study. The 3-year survival rate in patients with unknown stage is 56% which is slightly lower than those in stages II and III. Incomplete FIGO cancer staging may be associated with the socio-demographic and clinical characteristics of the patients, which may reflect a high proportion of patients with advanced stage at diagnosis. 

In this study, treatment has been classified as surgery only, radiation and/or chemotherapy, surgery combined RT and/or CT, and Palliative treatment. The results show that treatment types other than surgery only are independently associated with shortened survival and low 5-year survival rates as compared to patients who underwent surgery only. The treatment of cervical cancer depends on many factors including the type, stage of cancer, and possible side effects. Surgery may generally be given alone for the early stages of cervical cancer. The combination of radiation and/or chemotherapy and surgery combined RT and/or CT are recommended for more advanced disease (Sadalla et al., 2015; Wu et al., 2017). When patients with cervical cancer have distant metastatic disease, treatments options are often limited to either chemo-radiation or palliative treatment. The results of the study also suggest that patients with surgery only have a prolonged survival time compared to patients with other treatment types. Our analysis shows a significant association between certain types of treatment and survival in cervical cancer. It is not correct to infer a causal relationship from out data, and this association may be based on causality. 

The frequency of hospital visits is discovered to be an important factor associated with survival in cervical cancer patients. Patients who visit the hospital more frequently have better chances of survival. The results in this study are compatible with the results of the studies conducted by DorjiTiensuwan (2018), which indicates that a patient in Bhutan with cardiovascular diseases who regularly paid a visit to a hospital has a better life expectancy for cardiovascular disease. 

This study has some limitations, which may have influenced the results of this study. The major limitation is that the data have been obtained from the recorded of Jigme Dorji Wangchuck National Referral Hospital, which may not be generalizable to other areas. Additionally, the lack of some important risk factors may influence the accuracy of estimated survival and limit the effects of unrevealed significant prognostic factors. However, the best result could be achieved, if a greater number of patients were included with extra comprehensive variables such as current smoking habit, age at marriage, age at giving first birth, the number of partners, history of diseases, as well as histopathologic tumor characteristics. As the HPV infection vaccination program in Bhutan has been operating for more than 10 years and the 2019-2023 strategic plan for cervical cancer prevention and control has been recently implemented, a prospective follow up study could be conducted by incorporating prognostic and predictive factors to evaluate the impact in the short and long term of overall management including vaccination, screening and treatment.

In conclusion, Cervical cancer remains a significant public health problem in many low- and middle-income countries. Bhutan still has a high prevalence of cervical cancer. The present study shows that the overall probability of survival at 5 years among cervical cancer patients treated in Jigme Dorji Wangchuck National Referral Hospital between 2014 and 2019 is 55.4%, which is significantly lower compared to middle- and high-income countries. Risk factors affecting the life expectancy of cervical cancer patients also include age group, FIGO stage, type of treatments, treatment center, and frequency of hospital visits. Late diagnosis of cervical cancer appears to be mainly associated with a higher risk of dying from cervical cancer. In an effort to reduce the burden of cervical cancer, collaboration between the Royal Government of Bhutan, private organizations and local communities is critical to achieve the greatest impact of prevention and control programs. This includes promoting and developing cervical cancer awareness among the public so that women adopt healthy lifestyles and early screening behaviors. Availability, accessibility, acceptability and quality of health services for the diagnosis and treatment of precancerous and invasive cancer lesions are also key components for the elimination of cervical cancer.

## Author Contribution Statement

Study conception and design: PS. UT.; Data collection: UT.; All authors reviewed, analyzed, and interpreted the results, drafted the manuscript, and approved the final version of the manuscript.
